# On realizing autonomous transport services in multi story buildings with doors and elevators

**DOI:** 10.3389/frobt.2025.1546894

**Published:** 2025-02-25

**Authors:** Paul Robert Schulze, Steffen Müller, Tristan Müller, Horst-Michael Gross

**Affiliations:** Neuroinformatics and Cognitive Robotics Lab, Department of Computer Science and Automation, Institute for Technical Informatics and Engineering Informatics, Technische Universität Ilmenau, Ilmenau, Germany

**Keywords:** elevator riding, door manipulation, door detection, differential drive, system solution

## Abstract

Mobile service robots for transportation tasks are usually restricted to a barrier-free environment where they can navigate freely. To enable the use of such assistive robots in existing buildings, the robot should be able to overcome closed doors independently and operate elevators with the interface designed for humans while being polite to passers-by. The integration of these required capabilities in an autonomous mobile service robot is explained using the example of a SCITOS G5 robot equipped with differential drive and a Kinova Gen II arm with 7 DoF. This robot also defines the framework conditions with certain limitations in terms of maneuverability and perceptual abilities. Results of field tests with that robot in an elderly care facility as well as in a university office building are shown, where it performed transportation and messaging tasks. We also report on the success rates achieved and highlight the main problems we have encountered and dicsuss open issues.

## 1 Introduction

For years the development of socially assistive robots for home and also public applications has yielded promising user studies with positive expectations, but the lack of appearance of such systems on the market paints a different picture. One reason might be incisive boundary conditions for the robots’ deployment. Environments needed to be barrier-free, otherwise an expensive integration into the buildings’ infrastructure is necessary, or the robots would need human assistance of their own.

Against this background, the aim of our research project RobInCare^1^ (robots in care) was to enable robots to overcome the reason for impediment by developing respective capabilities to use an elevator and open closed doors on their own. The functionality is realized in our layered architecture Gross et al. (2012), which allows to integrate the new capabilities into other applications of our mobile service robots easily.

**FIGURE 1:**
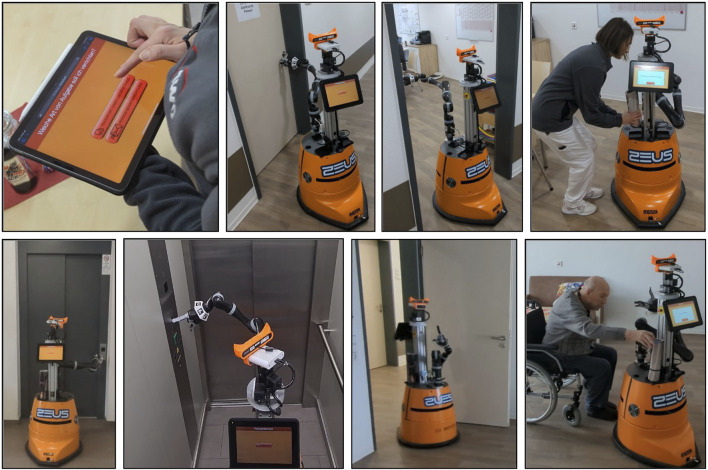
Robot performing a transport task in an elderly care facility; (from left to right, top to bottom) (1) task is scheduled via web interface, (2–4) robot opens and passes door to receive the goods, (5–7) robot has to ride an elevator on its way to the target person, (8) robot contacts the target person and finishes the transport task.

In order to test and evaluate the realized capabilities, we have integrated the developed methods into a transport and messaging robot, which is used in our university on the one hand and in an elderly care facility on the other (see Figure 1). The latter consists of a three story building with 24 apartments and public rooms for social interaction.

This article serves as a summary of the individual methodology used for realization of autonomous door manipulation and floor switching capabilities by means of operating an elevator. It describes the integration of these new functionality into a service application, while minor changes, which were necessary to achieve a smooth operation in a populated environment, are highlighted. We report the concluding experimental results conducted at the end of our research project and identify the open issues, which need to be addressed in future work.

In the following, we discuss the state of the art regarding robotic systems able to autonomously ride the elevator and pass through closed doors. Then an overview of our robot and the methods used are presented. Finally, we describe the setup and outcome of the experiments we conducted in order to evaluate the robustness of the autonomous navigation capabilities.

## 2 Related work

To the best of our knowledge, there is no publication of a comparable robot system which deploys the detection and manipulation of closed doors and the ability to ride an elevator in a real-life application. Nevertheless, there is significant literature dealing with the individual skills.

### 2.1 Using the elevator

One of the main problems to be solved for such a capability is the recognition and localization of the buttons to be pressed. Chen et al. (2023) recently introduced an approach to detect elevator buttons by means of a YOLOv7 object detector in images, which is a well understood standard approach that offers little room for improvement. Alternatively, Zhu et al. (2020) use an eye in hand system with a two step network architecture. One subsystem functioning as the button detector, and a second one implements a character recognizer to interpret the labels of the buttons, which might be necessary for environments with excessively many floors. Besides, there are also classic image processing approaches. Nguyen et al. (2023) read displays and button labels by applying a seven segment mask after the detection of squares and circles in the image. Abdulla et al. (2016) describes a novel framework for multi-story navigation through the use of an elevator by a mobile robot. Using external landmarks for accurate positioning in front of the elevator door and button panel, it detects buttons using a depth camera and multiple stages of image filters. The work declares a 98% accuracy in button detections.

Independent of the method used, the handling of unavoidable remaining detection errors is the key to a robust deployment. Zhu et al. (2020) use a multi-camera system and a post processing of detections by means of soft-non-maximum suppression and outlier removal to reach higher accuracy. For executing the actual push action, most systems rely on off-the-shelf offline motion planning, which is not described any further. The analysis in said studies concludes with giving success rates of the pure button detection or at most report the success rate of pressing buttons with a robot (e.g., 96% Nguyen et al. (2023)). Data on implementation of the whole procedure of calling, embarking, and riding the elevator are rare. Shin et al. (2023) describe a complete system and report a real-world experiment with nine out of 10 successful button operations and 3/10 successful boardings, which shows that real-world deployment is still questionable. In our own previous work Müller et al. (2023b) (sumarized in 3.1.3), we also describe the realization of the whole process with other necessary recognition skills, like floor tracking and cabin state analysis. The complete procedure also includes fallback and error handling in case of a failed button press, or failed elevator boarding.

### 2.2 Door manipulation

Similar to the elevator problem, there are no reports of real-world applications involving door handling. Rather, a broad variety of lab experiments concentrate on individual aspects of the door manipulation. One can divide the problem in the detection part and the actual manipulation. Image-based door detection is predominant among the approaches found. Arduengo et al. Arduengo et al. (2021) for example, apply a YOLO detector for the doors and use the depth image for identification of the door’s plane, and afterwards deviating points inside the door rectangle are interpreted as the door handle. The utilization of depth image data and the fitting of geometric primitives is a standard approach for analyzing the door’s exact location and opening angle. In contrast, we suggest to use a computationally less expensive approach based on lidar range scans Müller et al. (2023a).

On the other hand, there is the actual manipulation of the door. New approaches are concentrating on learning the opening process by means of imitation and reinforcement learning Sun et al. (2021); Welschehold et al. (2017); Ito et al. (2022). These may be capable of adapting to open new doors but at the cost of excessive training in real world as well as in simulation. Traditionally, in contrast there are many classic approaches, which categorize the door’s state and specify respective heuristics for processing these situations. For instance, Jang et al. (2023) use a graph of distinguishable situations and an $A^{*}$ planner to define a solving sequence of actions.

Often generic solutions are proposed that intend to work on arbitrary unseen doors, but this is not necessary for a deployment in a given environment. Incorporation of prior knowledge regarding specifics of the doors encountered helps to make an approach resilient. Experience from previous trials can also be taken into account when calculating door parameters for the next trial with a similar door model Arduengo et al. (2021).

Another crucial limitation that we have with our robot system compared to other solutions is the differential drive. While robots with a holonomic drive Arduengo et al. (2021); Welschehold et al. (2017); Ito et al. (2022); Stoyanov et al. (2023) can easily compensate the limited range of the robotic arm by moving sideways during the execution of the opening arc movements with hand on the handle, non-holonomic robots Karayiannidis et al. (2012); Endres et al. (2013); Sun et al. (2021) in literature often handle only a subset of the complete spectrum of door states neglecting the difficult cases.

Our previous paper Müller T. et al. (2023) (recapitulated in 3.1.4) describes the advantages and disadvantages of current approaches in detail and presents our own solution on a differential drive robot, which consists of a predefined sequence of manipulation strategies. These are based on prior knowledge of door geometry, which can be easily acquired when installing the system and therefore lead to a comparatively simple transfer to new application environments.

## 3 Materials and methods

### 3.1 System overview

#### 3.1.1 Robot platform

**FIGURE 2:**
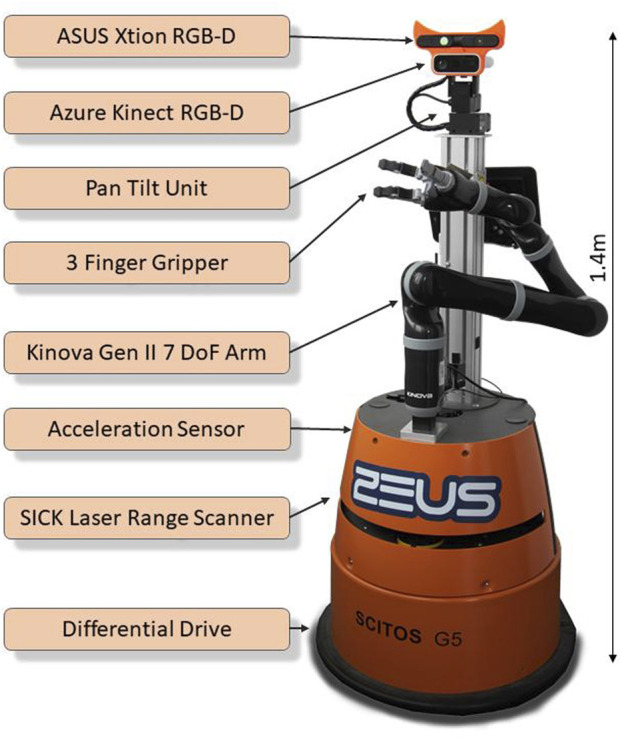
Used robot platform (Scitos G5 by Metralabs) as also seen in Müller et al. (2023b).

The methods for opening doors originally have been developed on a TIAGo robot with an under-articulated five-finger hand as end effector. Although successful door manipulation was possible with that robot, we later transferred the methods to a Scitos G5 robot by Metralabs, which is equipped with a Kinova Gen II 7 DoF arm. Figure 2 shows the robot with its main components used. The platform has a differential drive, which limits its maneuverability drastically compared to omnidirectional drives. This explicitly had to be considered during the door opening movement sequences. For perception the robot has two SICK Lidar sensors at a height of 40 cm, one being oriented forward and one backward. Furthermore, we use an Azure Kinect RGB-D camera on a pan-tilt unit on top of the robot to detect buttons when operating the elevator and to map the doors. An additional ASUS Xtion depth camera is used for obstacle mapping in order to ensure a safe operation of the arm. The active stereo approach of this device yields fewer artifacts in the point cloud than the time-of-flight Kinect camera. The built-in accelerometer of the robot platform is used to estimate the vertical movement in the elevator. Additionally it is equipped with a touch display for user interaction.

#### 3.1.2 System architecture

**FIGURE 3:**
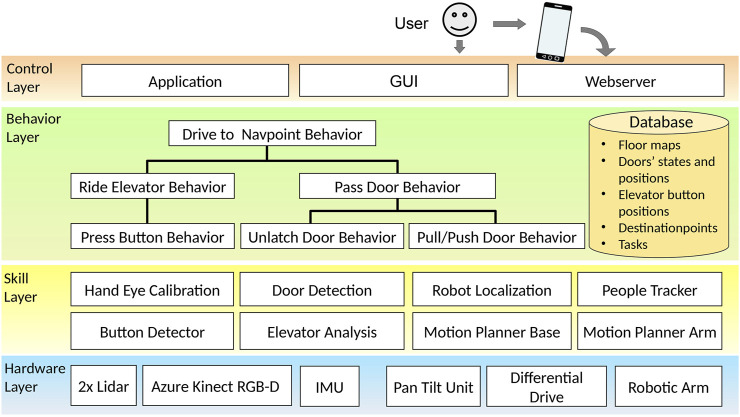
System architecture consisting of the fundamental skills necessary to ride elevators autonomously and open doors on the way.

We followed the layered architecture of Gross et al. (2012) when designing the modular software for our demonstrator. The implementation uses the robotic framework MIRA^2^, and our architecture depicted in Figure 3 is organized as follows: At the top is the *Control Layer*, which manages the interaction with users and the control flow. Here, the robot hosts a website for scheduling transportation or messaging tasks, which are stored in a local database on the platform. Having everything on board is crucial for the robot, as the WiFi connection might be interrupted inside the elevator and when switching access points on the way through the building. A user therefore can either interact with the robot via mobile devices to call the robot remotely or via the GUI displayed on the robot’s touch display.

In the *Behavior Layer*, we developed a hierarchy of reusable control loops that are activated exclusively one after the other. These behaviors make use of basic perception and action capabilities that are implemented as real-time processing units of the *Skill Layer*, which operate in parallel. Finally, the sensors and actors are accessed through the *Hardware Layer*.

**FIGURE 4:**
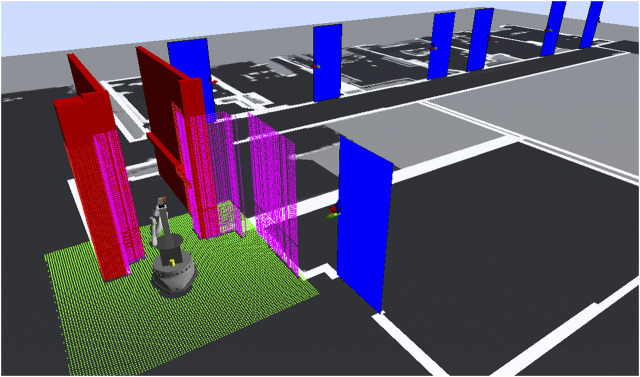
This figure illustrates the models used for safe navigation and manipulation. Navigation uses the 2D occupancy grid map (black and white on the floor). The arm motion planner relies on a 3d local collision map which combines data of various sources. The dots represent occupied voxels (green is the ground plane, pink the laser detections projected upwards, red static collision objects). The red boxes show the manually defined geometry of static objects. The blue door’s states are updated when seen in the lidar scan and are added to the 3d local map as planes if in range. Additionally, the pointcloud of the depth camera is integrated in the 3d local map as well.

For navigation, we use a hybrid prerecorded map of the building. Each topological section (floor) consists of a 2D occupancy grid defining the navigable areas, the location of doors and their properties (for example, the width of the door, or its opening direction), the location of button panels for interacting with the elevators, and a series of destination points that can be selected in the tasks by the users. For safe obstacle avoidance during manipulation, a local 3D voxel map is generated in real time, using the live point cloud from the ASUS Xtion camera and vertical walls generated from the horizontal lidar scans, which comprise areas that are not covered by the camera. This is illustrated in Figure 4, with the robot positioned in front of an elevator. Furthermore, for non-vertical structures such as handrails, a 3D representation of obstacles is added manually and also stored in the global maps. The local collision map has a cell resolution of 2 cm, while in each cell, the exact position of the obstacles are stored. For collision testing with the robot geometry during motion planning the local collision map is processed with a distance transform in order to yield distances to the closest obstacle in each of the grid cells.

The mapping process of the door properties is semi-automated. To this end, a YOLOv5 detector network trained on the dataset of Arduengo et al. (2021) identifies doors and detects door handles in the RGB images during the initial mapping of the building. The point cloud of the depth camera is then used to analyze the properties of the doors in the detected bounding boxes. Points are projected on the ground plane where lines are fitted to calculate the distance between the door and the enclosing walls. This yields the opening direction of the door. In a similar way, the occurrences of buttons for the elevator are recorded during mapping, while the geometry and semantics of the button panels are annotated manually. With the help of this automation, the deployment in a three-story building was completed in just a few hours, although there is certainly still potential for further automation of these annotation tasks.

Based on the data in the maps, we implemented the fundamental *Drive To Navpoint Behavior* (see Figure 3), which checks whether the destination is on the same floor and activates the *Ride Elevator Behavior* if necessary. For navigation to destinations on the current floor, the planned path is intersected with the lines of closed doors in order to trigger the *Pass Door Behavior*, if necessary, which analyzes the actual state of the door and triggers corresponding actions to open it.

The 2D navigation skills of the robot are based on an $E^{*}$ metric planner and an evolutionary local motion planner, described in detail in (Müller et al., 2017).

For localizing the robot in the environment, a Monte Carlo approach (Fox, 2001) is used, which relies on matching the lidar scans against the occupancy maps. In a previous project (Gross et al., 2015) using the same localization method on a similar differential drive robot, the accuracy of the localization has been evaluated to be within 7 cm. Unfortunately, this limited accuracy also affects the position of modeled obstacles. In order to reach buttons and door handles with the arm without collisions, a more accurate localization is required. To that end, we use the detections of button panels and door keypoints as additional observation updates for the particle filter-based localization, such that the deviation due to the coarse map resolution of 5 cm can be reduced when the robot is facing a door or button.

#### 3.1.3 Operating the elevator

**FIGURE 5:**
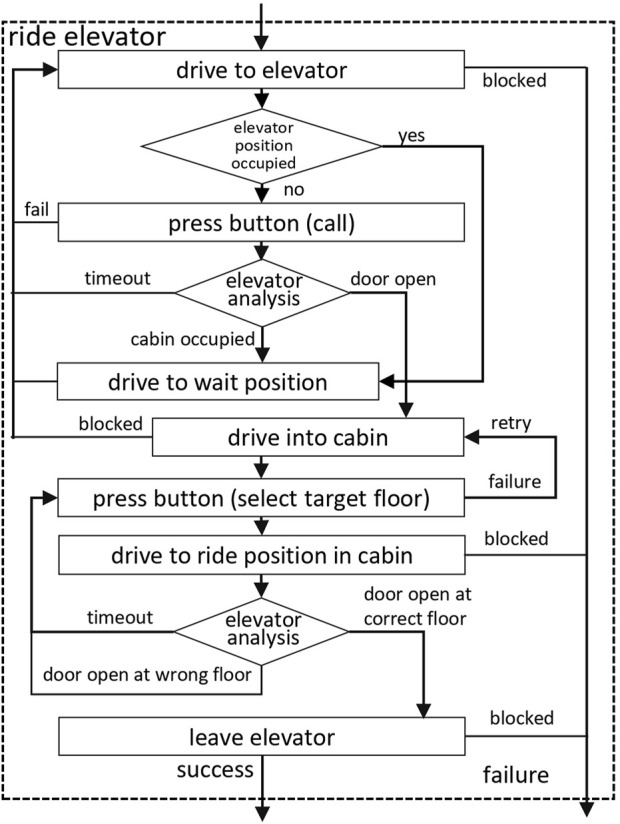
Flow-diagram of the ride elevator behavior algorithm.

The *Ride Elevator Behavior* (see Figure 5 for a coarse sequence of the algorithm) implements a sequence of actions in order to use the elevator. The workflow is basically the same as described in Müller et al. (2023b), except that a handling of traffic in front of a lift to be used is added. First, the area in front of the elevator is analyzed for other people who might be waiting for a ride. They get precedence, and in this case the robot drives to a waiting position. Wengefeld et al. (2019) gives an overview of the multi-modal people detection and tracking system we use on the robot.

Once the way is free, the robot places itself in front of the elevator door and activates the *Press Button Behavior* to call the elevator. Then it observes the state of the cabin door and brings its arm back to a safe home position, which is used for driving. The state of the elevator door is determined in the lidar range scan by checking the width of the free gap on the known line of the door. When the door opens, the cabin is analyzed for people inside, which might leave the cabin. If there are passengers in the elevator, the robot gives way and starts over with the call elevator procedure afterwards. In the case of an empty cabin, the robot enters the elevator as quickly as possible, which was a tough challenge as the door only stays open for 6 s.

Inside the elevator, the *Press Button Behavior* is used again in order to select the destination floor, and the robot immediately retracts its arm and moves to a waiting pose in front of the door, as the time for exiting is also limited. In the meantime, the robot keeps track of the current floor the elevator is on by measuring the time between the peaks of vertical acceleration and deceleration (see Müller et al. (2023b) for a more detailed analysis of this solution).

When the elevator stops at the target floor, the robot leaves the cabin and communicates to potential passengers waiting in front, that it needs space to maneuver. In case the elevator stops at another floor, the robot tells waiting people, that it is occupying the elevator and wants to go alone in order to prevent any contact and blocking of passengers, and starts over with the dial target floor procedure in case a wrong button has been pressed, since the robot cannot distinguish the reason for the stop at the unintended floor. This repetition of dialing is also triggered, if the elevator does not move after a button has been pushed.

**FIGURE 6:**
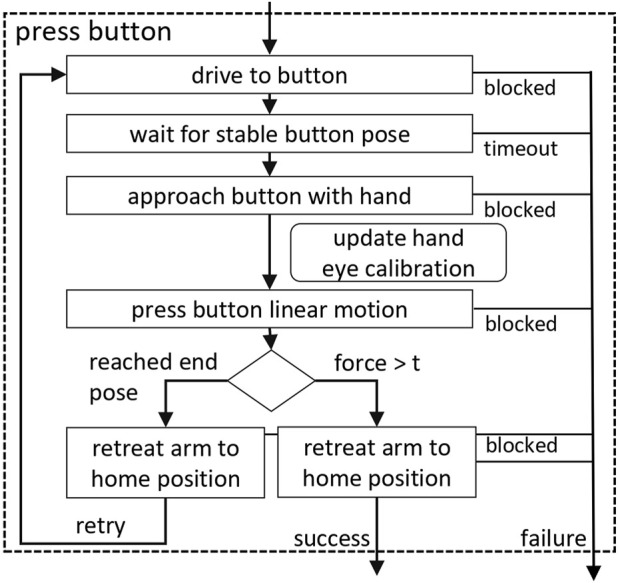
Flow-diagram of the Press Button Behavior as also seen in Müller et al. (2023b).

The *Press Button Behavior* (see Figure 6 for a broad procedure of the algorithm) is relying on a Faster-RCNN detector able to localize 16 button classes in the color image. The resulting bounding boxes are used for a lookup in the depth image to obtain 3D positions and surface normals of the individual buttons, which are matched against the previously known button patterns on the mapped panels. The number of distinguishable buttons limits the applicability to buildings with reasonable number of floors. Nonetheless, more than 10 floors are also possible due to the panel matching as described in (Müller et al., 2023b). The matching of complete panels to incomplete detections allows to compensate for unrecognized and misclassified buttons and therefore correct buttons can be pressed even if they cannot be detected individually. A detailed evaluation of the detection accuracy can be found in (Müller et al., 2023b).

Having the 3D pose of the target buttons, the motion planner (Müller et al., 2021) is used to execute a preparing approach movement that brings a finger in front of the button. This is followed by a linear push movement, that is terminated by a force feedback event.

Since we do not use an eye in hand setup but determine the target position for the push movement in camera images, the robot model needs to be carefully calibrated against the movable camera. To that end, the end effector of the arm is labeled with a set of ArUco markers (see Figure 10 right), which allows to calculate the exact position of the fingers in camera coordinates at runtime, provided they are visible. This is used to compensate for deviations between the robot model used for planning the motion and the real position of the robot arm with respect to the camera and increases the hit rate drastically during the push movement. The marker and button detections runs with a frequency of 5 Hz on a 1280 by 720 pixel image. The accuracy of the marker localization in lateral direction usually is in the range of the pixel resolution, while depth estimation from 2d image data is more error prone. Fortunately, the accuracy of the targets’ distance in the direction of movement when pressing the buttons plays a subordinate role.

#### 3.1.4 Opening doors

**FIGURE 7:**
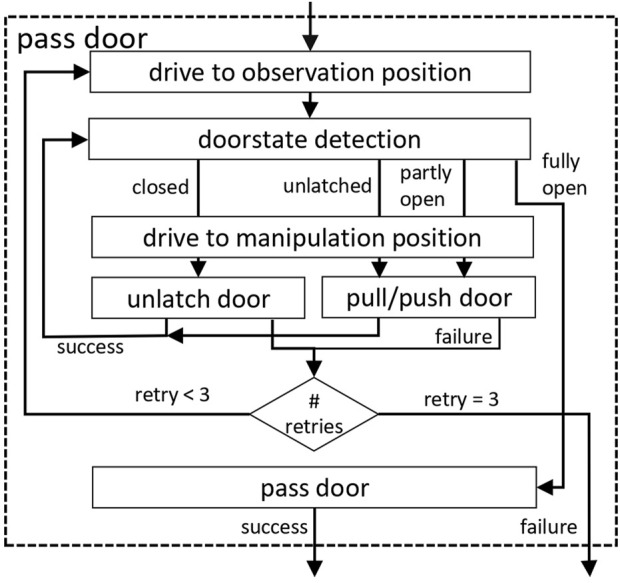
Flow-diagram of the pass door behavior.

Opening and passing through doors is governed by the *Pass Door Behavior* (see Figure 7), that initially approaches a coarse observation position, from where the door’s current state and exact position is analyzed. Unlike many other solutions, our door analysis is based on lidar range scanning and not on image processing. This saves computational resources and is robust enough, as an analysis of the position accuracy for reaching the door handle shows (see Section 4.1.1). A detailed description of the door localization in range scans using a 1D CNN can be found in (Müller et al., 2023a) The detection of the door runs at the rate of the lidar scans, which is 15 Hz. This additionally increases the robustness of the estimated position of the door.

**FIGURE 8:**
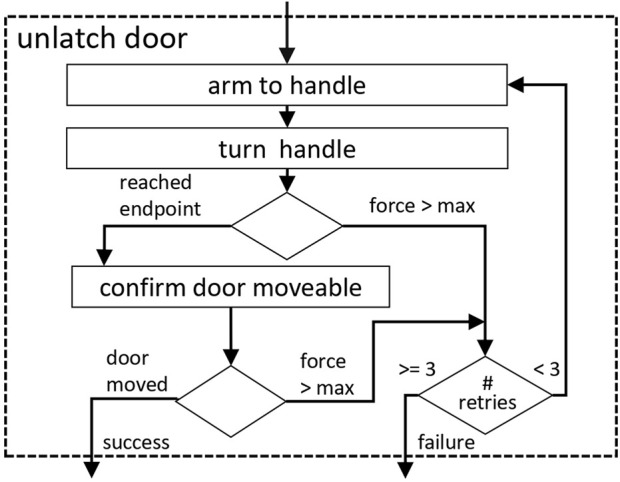
Flow-diagram of the unlatch door behavior.

Depending on the observed opening state of the door, specialized subordinate behaviors (unlatch door, pull/push door, or simply pass through the open door) are activated, each of which is responsible for bringing the door to the next more open state (closed → unlatched → partly open → fully open). Figure 8 shows the procedure for unlatching a closed door for example,.

**FIGURE 9:**
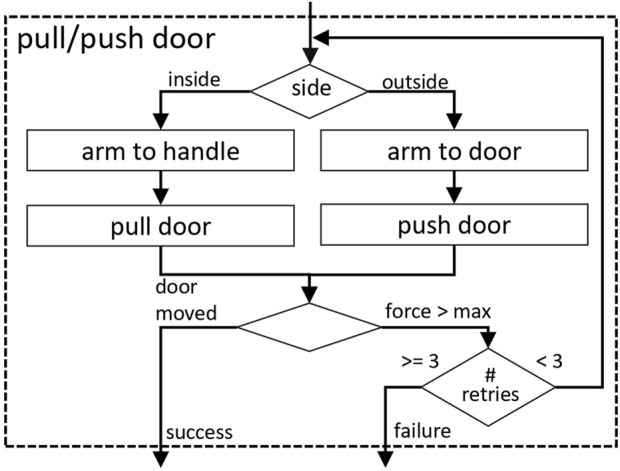
Flow-diagram of the pull/push door behavior.

For these operations, it depends on the side of the door and the opening direction whether to grasp the door handle for pulling or only to push the door on its edge or face with the open hand (see Figure 9). All movement sequences have been designed by hand and consist of a series of intermediate navigation points for both the robot base and the arm, which are defined in the door reference frame and scaled with the known door width. In this way, individual deviations in the robot’s position can be compensated. One aspect of importance is the fact that coordinated movements of the robot’s base and the arm are necessary because of the limited reach of the arm when the door swings open. This coordinated movement is achieved by using a closed loop motion planner for the arm (Müller et al., 2021), which runs at a frequency of 4 Hz. By means of that, the slower motions of the base can be compensated for and the end effector trajectory with respect to the door can be achieved.

The limitations due to the differential drive (robot cannot move sideways) and the limited free space behind an open door make it impossible for our system to open a door swinging towards the robot without repositioning of the hand from the inside to the outside handle. In order to pull open a door to the fully open state with the end effector at the inside handle, due to the limited reach of the used arm, the robot base would need to follow the door in a circular movement ending up behind the open door. This, in most of the doors seen in our scenario is not possible because of restricted free space. Consequently, we have to let go the handle, when the robot is moving through the door. As we only have one manipulator onboard, the robot is prevented from opening doors with automatic closing mechanisms, since during reposition of the end effector and when passing the open door, the door cannot be locked in position.

Other external disturbances at the door, such as people opening the door from the other side, can be compensated by the supervising *Pass Door Behavior*. This triggers retry mechanisms for the individual strategies. A repetition is also triggered if the gripper slips off the handle while manipulating the door. This is recognized by monitoring the force and torque measurements at the end effector.

If the door could not be opened after three retires or restarts of the behaviors, it is highly likely, that the robot is falsely localized or the door might be locked and required human intervention. More details regarding the door manipulation strategies and the *Pass Door Behavior* can be found in Müller T. et al. (2023).

#### 3.1.5 Limitations

Unlike other publications, our system does not aim to reactively handle unseen doors. Rather, we intended to realize a reliable solution for deploying a transportation service in a predefined operation environment. Although we rely on a previously mapped environment (doors and elevator), achieving a robust autonomous transport service managing the doors and elevators is still challenging. The robot needs a high degree of introspection and needs to be able to recognize deviations from the expected behavior, especially during the manipulation activities.

For example, collision detection is implemented based on the forces measured at the end effector. If a certain threshold is exceeded, the system halts. Here, it is difficult to distinguish intended interaction forces from accidental contact. In contact situations, ethical questions arise, such as whether the robot is allowed to retry moving or whether human intervention is required to recover from such situations.

Another situation is in the elevator. The robot tells people that it occupies the cabin and wants to go alone in order to prevent any contact and blocking of passengers. Nevertheless, nothing prevents people from entering the elevator and interfering with the robot’s behavior. If people push the robot around, it may get stuck and require for human intervention to recover.

In conclusion, we have tried our best to detect and handle as many exceptional situations as possible, but due to a lack of recognition capabilities, there will always be situations that the robot cannot handle autonomously. In the evaluation section we will give examples that have occurred.

## 4 Results and discussion

### 4.1 Evaluation

#### 4.1.1 Analysis of grasp pipeline accuracy

All intermediate grasp poses of the door manipulation strategies are designed by hand and might depend on the exact situation at the site of development. Nevertheless, each individual door handle should be reached consistently in order to achieve a reasonable success rate for opening the door.

Therefore, we first tested the positioning stability of the entire pipeline of the integrated system at once. So the influence of the door localization in the range scan, the robot platform localization and navigation capabilities, and the accuracy of the execution of the arm motion contribute to the final end effector position with respect to the door handle.

**FIGURE 10:**
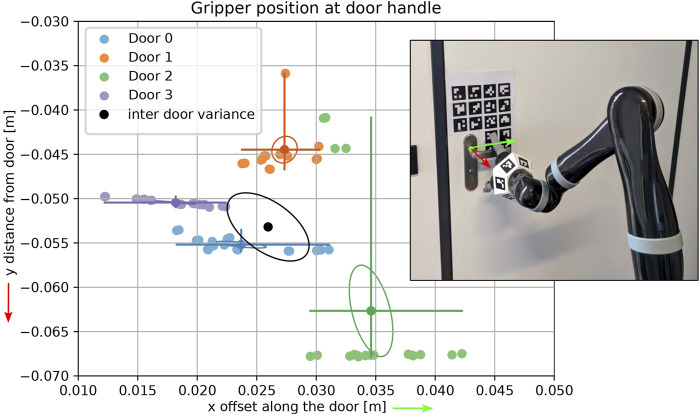
Distribution of end effector positions for multiple trials of approaching and grasping the door handle of four different doors. The colored lines mark the maximal deviation on both axes. With (0, 0) marking the handle pivot point.

In order to measure the consistency of the end effector positioning, we utilized ArUco markers, which we placed beside the door handle in an exact reference position to the axis of the handle. For four different doors of different kinds (since the evaluation environment did not offer more variety), we used these visually recognizable markers and the calibration markers on the arm (see Figure 10 right) to record the distribution of the relative position of the end effector to the door handle. For each of the 15 trials per door, the robot first was approaching the manipulation position with the robots’ base and conducted a grasping motion to approach the handle afterwards. When the target position on the door handle has been reached, the positions have been recorded. Since the necessary door analysis is carried out in lidar data only, the additional visual markers have no influence on the results, but they do allow to measure the relative position precisely.

Figure 10 shows the intra- and inter-door variance of the four measured doors, with the handle pivot point in (0.0;0.0) . The intra-door variance describes the distribution of the resulting grasp poses for one individual door and therefore reflects the stability of the grasping pipeline in application conditions. The inter-door variance, on the other hand, mainly reflects the influence of the door analysis and localization network. The exact localization outputs for the door hinge and handle depend on the profile of the actual closed door, seen in the lidar scan. Since different doors have differently shaped door frames, a bigger inter-door standard deviation of 5 mm in x -direction, 9 mm in y -direction (see the black ellipsis in Figure 10) results. For application in our scenario, this is of minor relevance, because it can be compensated for with a door specific correction of the grasp target pose at the handle.

The average intra-door standard deviation over all trials (3.2 mm in x -direction and 3.5 mm in y -direction) confirms that the stability of the localization method and the navigation and manipulation pipeline together is sufficient for a robust manipulation of the doors. The actual minimum and maximum grasping position lies in a range of about 1.5 cm around the target at the handle for a given door. The horizontal and vertical deviation of the targeted point on the handle of all trials is about 3 cm, which is roughly the area we need to hit in order to apply sufficient torque on the handle pivot point, to unlatch the door (not too close to the axis of the handle) without slipping off the other end of the handle. These results support the hypothesis that the system will easily generalize to other doors.

#### 4.1.2 Stability of the deployed system

Since our approach is one of the first known autonomous robotic systems to perform a series of combined door openings and elevator uses, the evaluation of our approach (see Table 1) might be able to function as a coarse reference metric for future systems. We first take a look at the system as a whole, then go into detail on the specific behaviors of elevator riding and door manipulation, and conclude with a summary of problems encountered during testing.

**TABLE 1:** Evaluation of the presented system.

Location	Tasks	Quote	Duration [min:sec]		
			Min	Avg	Max
Full system					
AWO	79	88,6%	01:13	05:47	13:47
Zuse	40	80,0%	02:15	06:37	12:03
Elevator riding					
AWO	44	93,2%	01:39	02:07	05:23
Zuse	25	88,0%	01:26	02:48	06:14
Door manipulation					
AWO	115	94,8%	01:06	01:45	04:30
Zuse	42	88,1%	01:06	02:07	06:03

##### 4.1.2.1 Task and duration evaluation

**FIGURE 11:**
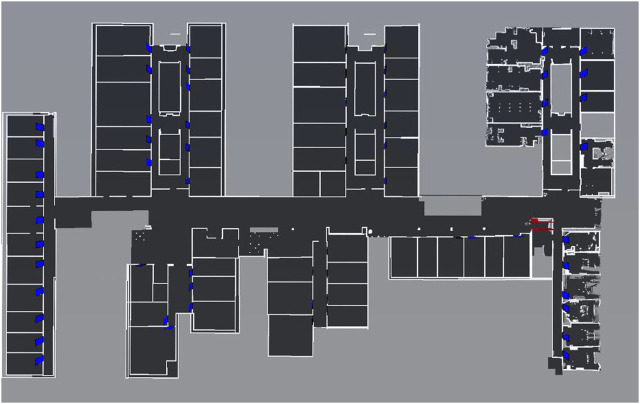
This figure shows one floor map of our three-storey university building with the 3D collision objects (elevator and doors) as overlay on the 2D occupancy grid map.

The robot system was fully tested at two different locations. The first one is an elderly care facility of the workers welfare association (short ger.: *AWO*) and the second one is our office building named after Konrad *Zuse*. A floor map can be seen in Figure 11 with the doors and elevator included. In both buildings, the robot had to execute a total of 119 transportation and notification tasks. In a transportation task, the robot has to drive from the home position to a pick-up location and afterwards to the destination, where the object is delivered. For messaging tasks, the robot simply drives to the destination and waits for a person to be notified. Once the people tracker recognizes a human, the message is presented to the person.

During the test campaign, each task comprised one or more door opening procedures and up to two elevator uses. The robot was accompanied by a supervisor who noted exceptional behavior and recovered the system if necessary.

79 of the tasks were performed at the AWO facility with an overall success rate of 88.6% , leading to a failure in 9 cases. We had a slightly lower success rate of 80.0% for the tests in the Zuse building. The average time to successfully complete a task stays between 5 and 7 min and is mainly influenced by the distance to drive, the number of doors to open and floor changes required. The minimum time span of 1–2 min arises from a task in which the target is on the same floor behind just one closed door. Whereas the maximum durations of the 10 min can be traced back to situations where many retries in the door manipulation strategies were necessary.

Further, we evaluated each use of the elevator separately. In the AWO environment 41 out of 44 elevator rides were successfully completed, while in the Zuse building 22 of 25 rides worked well. This yields a success rate of 93.2% and 88.0% respectively.

On average, the robot needs around 2–3 min for an elevator ride, with 1:30 min being the minimum time span to change floors. One fact that must be taken into consideration is other people using the elevator, causing the robot to wait for the next free cabin. Considering necessary retries while waiting and traveling to an intermediate floor, the maximum time period the robot took was less than 7 min.

The door opening procedure was evaluated at the end of Table 1 with 115 tasks in the AWO environment and 42 attempts in the Zuse building. Our approach successfully opened 94.8% of the doors at AWO and 88.1% in the office building. The varying success rate in the two buildings is due to various problems, ranging from localization errors to problems with force thresholds for detecting the end points of movements (as seen in Figure 8 or Figure 9). The latter had the largest impact on the duration, as each new attempt to unlatch the handle adds at least 20 s to the total time. The minimum time required to open a door is about 1 minute, independent of the location. We can therefore assume that different buildings generally have no influence on the duration of the pure manipulation.

##### 4.1.2.2 Failure reasoning

The main failures can be divided into three classes: i) *Localization*, ii) *Detection*, and iii) *Obstacle Perception and Motion planning*.

Starting with the localization, one of the major differences between the two buildings is their internal structure. The Zuse building has long hallways with few features for localization on the one hand and wide open spaces on the other. In addition, the robot often has to cover distances of up to 100 m. In contrast, the AWO facility is more feature rich with clear edges and junctions as well as much shorter hallways, like in a home environment. Therefore, the localization of the robot in its map at the AWO facility is more robust, which is reflected in the overall success rates.

Although we extended the Monte Carlo localization approach to accurately localize the robot with respect to observed doors and buttons, sometimes the robot could not recover from global localization errors. The reason for this is that these additional observations are only active when such targets are nearby and also require a good prior estimation of the robot’s position to work (correct sections of the range scan need to be extracted for analysis). Incorrect positioning in the map and thus a following localization error of door features or button positions can lead to subsequent misbehavior without a direct indication for the robot to recognize them. For example, a wrong position may prevent the robot from recognizing the elevator door to be opened, resulting in an infinite loop of attempts to call the elevator. It finally needed human intervention in such situations as occured two times in the Zuse building. Thus, one of the main disadvantages we will have to overcome in the future is the precise self localization in large, wide-open and unstructured areas.

A second problem related to the localization is caused by false localization of buttons and door features. If the erroneous detections are used to adjust the robot’s global pose in the map, it might virtually be moved into static obstacles. Then the robot gets stuck during navigation or manipulation as it spuriously believes to be in collision. It can no longer safely recover from this situation, as localization updates require the robot to move in order to obtain new independent measurements. Therefore, finally human intervention is needed. This problem occured in the AWO elevator, which has very little space left for manouvering (less than 4 cm at the robots tail when turning).

Concerning the problems related to the detection and localization of door keypoints, we observe an instability of the predicted positions when the robot moves during operation. Since the door analysis runs continuously during door manipulation in order to keep track of the opening angle, the actual arc trajectory of the hand may shift with the localization of the door hinge used as the pivot point. When the arm motion planner reacts to that changing target trajectory, contact forces can exceed thresholds and the opening process is interrupted, requiring a retry. We aborted the operation after the third attempt of each stage of the procedure. Such erroneous localization of door features occurred mainly when the robot was close to the door or in between the door frame. These are poses that were not part of the dataset of lidar scans used to train the detector. We tried to make the detection more robust in these situations by verifying the location of the door’s hinge and opening angle using a line fitting heuristic. Nevertheless, uncertainties remain when there are contradictory predictions for the position of the door, and exceptional situations could not be completely avoided.

The last category of problems is related to the limited perception of the environment and a lack of accuracy in the internal representation of the robot’s geometry in relation to its real shape. Due to the limited precision of depth perception and difficult conditions, e.g., in the elevator where mirrors and reflective surfaces are prevalent, the robot can come into unintentional contact with obstacles. This resulted in one of the unsuccessful elevator rides. Also, the non-deterministic nature of the used motion planner is sometimes causing problems, when occasionally unconventional solutions for a movement trajectory are produced that have not been seen during development. These alternative solutions can bring the arm into unforeseen configurations, which result in inadequate poses for succeeding steps, due to unfavorable joint limits. Further restrictions in the definition of the desired movements must be introduced in order to eliminate these circumstances in future.

To summarize, there are many interdependent subsystems in our approach that can fail and the success rate for complete task execution is the product of the individual ones. Although the individual parts can be made more robust, there will always be critical situations that the robot cannot perceive with its limited sensory capabilities alone. This requires a safe hardware design to make it usable anyway.

## 5 Conclusion

In this article, we presented a first integrated solution for a service robot operating in populated buildings with elevators and closed doors. The long-term real-world application tests revealed that the system basically operates autonomously thanks to its safe hardware design. Although task completion still fails in 14% of cases when doors and elevators are on the way, the robot cannot harm any people and can be left running unattended. We identified aspects for future improvement in our methodology, but ultimately there will always be situations that cannot be anticipated and require human intervention due to the robot’s limited introspective and perception capabilities. In the latter, we see the greatest potential for optimization in the future.

## Data Availability

The original contributions presented in the study are included in the article/Supplementary Material, further inquiries can be directed to the corresponding author.

## Footnotes

1
https://www.robincare.de/

2
https://www.mira-project.org
